# The Lived Experience of Crossing the Road When You Have Developmental Coordination Disorder (DCD): The Perspectives of Parents of Children With DCD and Adults With DCD

**DOI:** 10.3389/fpsyg.2020.587042

**Published:** 2020-11-19

**Authors:** Kate Wilmut, Catherine Purcell

**Affiliations:** ^1^Perception and Motion Analysis Lab, Department of Psychology, Health and Professional Development, Faculty of Health and Life Sciences, Oxford Brookes University, Oxford, United Kingdom; ^2^School of Healthcare Sciences, College of Biomedical and Life Sciences, Cardiff University, Cardiff, United Kingdom

**Keywords:** pedestrians, Developmental Coordination Disorder, co-occurrences, risky behavior, road crossing

## Abstract

Pedestrians are one of the most vulnerable groups at the roadside, furthermore, previous research has demonstrated perceptual-motor limitations in individuals with DCD which may put these individuals at even more at risk in the context of road crossing. However, it is unclear whether this is the lived experience of these individuals at the roadside. Furthermore, difficulties with road crossing and safety have been found in other neurodevelopmental disorders but the impact this might have on an individual with co-occurring difficulties is unknown. Therefore, we utilized a questionnaire to survey the lived experience of adults with DCD and parents of children with DCD with the specific objectives of describing behaviors exhibited by adults and children with DCD (the latter reported by parents) at the roadside and to determine the how these individuals perceive road crossing actions. For each of these we compared different co-occurrence groups. We also had one final objective which was not focused on road crossing but more on the general perception of accidents and unrealistic optimism. Individuals with co-occurrences which have previously been linked to unsafe crossing behaviors (i.e., ADHD, ASD, and LD) reported a greater regularity of dangerous looking behavior (forgetting to look, running without looking) and visibility (crossing between cars, crossing when you can’t see), these adults and the parents of these children were seemingly aware of the risky nature of these behaviors. When asked “why” crossing ability might be different, perceptual and motor difficulties alongside heightened awareness of risk and lowered awareness of risk were all cited by participants. Unrealistic optimism was not an explanation for the risky behavior in adults with DCD and in fact, these adults demonstrated a clear understanding of the likelihood of accidents. The findings of this study suggest that road crossing is perceived to be more challenging for both children and adults with DCD and this needs to be taken into account when considering remediation for this group.

## Introduction

According to the World Health Organization, more than 1.25 million people die each year as a result of preventable road traffic accidents (World Health Organization, 2018). Furthermore, tens of millions of people are injured or disabled on the world’s roads each year, with a high proportion of these people being pedestrians, i.e., in 2017 a total of 21% of all people killed on roads were pedestrians (European Commision, 2018). Although there is no accurate record of the economic cost of pedestrian deaths alone, the estimated cost borne from road traffic accidents is £12bn per annum in Great Britain (Department for Transport, 2019), or between 1 and 2% of gross national product (World Health Organization, 2013).

Clearly road traffic accidents bear both an economical and societal cost and pedestrians are considered to be a vulnerable road user. However, a population who might be at an even greater risk at the roadside is children and adults with Developmental Coordination Disorder (DCD). DCD occurs in 2–20% of the general population (Blank et al., 2019) and is an idiopathic condition characterized by marked impairments in motor coordination that negatively impact on activities of daily living which persist into adulthood (Kirby et al., 2013). No direct statistics exist regarding the number of roadside accidents for this group, however, recent research from our lab has highlighted a potential heightened vulnerability in this population (Purcell et al., 2011, 2012, 2017; Purcell and Romijn, 2017). For example, the ability to detect a vehicle as approaching (looming sensitivity) is consistently poorer in primary school aged children with DCD (Purcell et al., 2012) with children with DCD failing to detect vehicles approaching at speeds in excess of 14 mph in laboratory conditions, compared to 20 mph in primary school aged typically developing children (Wann et al., 2011). Assuming a vehicle is approaching above visual-perceptual threshold, and can be seen as moving, the child must then make a judgment regarding time-to-contact (TTC), previous evidence suggests further immaturities in children with DCD in TTC tasks as compared to their typically developing peers (Purcell et al., 2011). The studies mentioned above considered the perceptual component of road crossing, however, the ability to safely cross a road is a perceptual-motor skill which involves coordination between a pedestrian’s perception of the approaching vehicle and their locomotive capability to execute the road crossing action. Previous evidence has demonstrated that children with DCD accept significantly shorter temporal crossing gaps as speed increases and tend to choose gaps which are too short given their walking speed (Purcell et al., 2017). Furthermore, although a DCD population was not included in their study, (Pitcairn and Edlmann, 2000) did find a correlation between poorer performance on a fine motor control task and errors in roadside perceptual judgments, both in typically developing children and adults. Taken together, the above program of research provides a clear indication that the perceptual-motor system in children with DCD may put them more at risk at the roadside. However, these limitations to the system could be overcome by: waiting a long time to cross; always using a signalized crossing and/or always crossing with another individual. These factors are difficult to determine in a lab based environment therefore, it is important to consider road crossing behavior and experience outside of a lab environment.

Two studies have attempted to explore the road crossing experiences of children with DCD. The first asked a small group of children with DCD to self-rate their perception of their ability and confidence in a road crossing environment and found no difference in either perceived crossing ability scores or confidence in their ability to execute a safe road crossing by themselves compared to their typically developing peers (Purcell, 2012). However, the children with DCD did perceive the task as significantly more dangerous. Likewise, a later study found no difference between children with DCD and their typical peers with regards to their self-reported knowledge of safe crossing places, their confidence in road crossing skills, their perceived road crossing ability, how often they felt they misjudged traffic gaps or having to wait a long time to cross (Purcell and Romijn, 2017). The mismatch here between the child’s perceived judgment of their ability and previously identified limitations of the perceptual-motor system in children with DCD is striking and would suggest that this could place these children at even greater risk as they have less refined perceptual-motor skills and don’t seem to recognize this limitation. These previous studies have made direct comparisons between how primary school aged children with DCD and their typically developing peers perceived their behaviors at the roadside (i.e., were they risky or not), but they didn’t ask participants to rate the occurrence of specific behaviors. This is an important distinction as children may not self-report exhibiting risky behavior but they may often exhibit a behavior which would normally be considered as risky. For this reason the current study goes one step further and asks about specific behaviors. In addition, we also include a sample of adults with DCD. Despite the paucity of current literature we do know that DCD is a lifelong condition and adults with DCD continue to experience difficulties throughout their adult life (Cousins and Smyth, 2003). Which includes difficulties which are key in a road crossing context such as visual motor integration (de Oliveira et al., 2014), gait variability (Du et al., 2015), and executive functioning (Tal Saban et al., 2014). Furthermore, whereas a child may often be accompanied at the roadside either by friends, siblings or a parent this is rarely true for adults. Therefore, describing the experiences of a group of adults with DCD is an important first step. The current study, therefore, describes the lived experience of crossing the road in a large sample of individuals with DCD and their parents.

One interesting finding raised by Purcell and Romijn (2017) in their questionnaire was that children with DCD rated the act of crossing the road as more dangerous than their typically developing peers. The idea of how dangerous an activity is, has been considered in typically developing children and adults, if we see an activity as not posing any danger we would approach it differently from an activity which is seen as dangerous. However, our perception of danger can be biased, one such bias which lessens the appreciation of risk is unrealistic optimism or the “it won’t happen to me” mentality (White et al., 2011; Shepperd et al., 2013). Studies which have demonstrated unrealistic optimism in typical adults include, but are not limited to, estimating the risk of a heart attack (Radcliffe and Klein, 2002), the risk of experiencing severe alcohol problems in the future (Dillard et al., 2006), women’s estimated risk for breast cancer (Waters et al., 2011), and smokers’ estimated risk of cancer (Ayanian and Cleary, 1999). Unrealistic optimism has also been considered in children (Sissons Joshi et al., 2017) with participants comparing the likelihood of common childhood accidents happening to them compared to their peers, children consistently stated that an accident was “less likely” to happen to them. Children cited reasons such as “heightened skill” or “lack of exposure” as reasons for why these accidents were less likely. Therefore, this study suggests that typically developing children are unrealistic about the likelihood of accidents and so might take risks without perceiving them as risks. Whether this unrealistic optimism extends into individuals with DCD is unclear, however, studies have demonstrated that unrealistic conditional optimism is responsive to factors such as controllability and personal experience (Helweg-Larsen and Shepperd, 2001) and so might not be as prevalent in individuals with DCD if they perceive themselves as less in control because of their motor difficulties or have had previous experience of such accidents. The important factor here is that evidence suggests that people are less likely to take precautions if they perceive their absolute risk as low (Floyd et al., 2000) and so understanding the perception of risk in this population is key.

A final consideration in the current study is co-occurrences with other neurodevelopmental disorders. Often research studies might exclude participants on the basis of additional neurodevelopmental disorders in order to be sure that their research is describing the effects of a single disorder rather than looking at something else. However, in terms of the lived experience, an individual with multiple neurodevelopmental disorders experience the effects of all of them simultaneously. Research has shown that children with Attention Deficit Hyperactivity Disorder (ADHD, ADD) are more at risk at the roadside as pedestrians (DiScala et al., 1998; Brook et al., 2006), are less concerned about risk (Farmer and Peterson, 1995; Mori and Peterson, 1995) and accept crossing gaps which leave them with small safety margins (Clancy et al., 2006; Stavrinos et al., 2011). Furthermore, children with Autism Spectrum Disorder (ASD) show a poor understanding of how to use traffic signals (Josman et al., 2008) and adults with ASD display different looking behavior compared to peers (Earl et al., 2016, 2018; Cowan et al., 2018). Finally children with learning disabilities (LD) have been found to demonstrate difficulties in identifying safe crossing places (Anastasia, 2010). Although the studies cited above widely ignore other neurodevelopmental disorders it does suggest that co-occurrence of multiple neurodevelopmental disorders might place an individual at an even heightened level of risk as a pedestrian.

The current studies primary aim was to describe and explore the lived experience of adults with DCD and parents of children with DCD at the roadside and to highlight factors which may influence this, with a specific focus on the impact of co-occurrences. Within this primary aim we had two distinct objectives which focused on road crossing, the first was to describe the behaviors adults with DCD and children with DCD (as reported by their parents) exhibit at the roadside. The second was to determine the how these individuals perceive their road crossing actions (using both closed questions and an open question). We also had one final objective which was not focused on road crossing but more on the general perception of accidents and unrealistic optimism. Some of the accidents that participants were asked about have a clear motor component and so for these we expected adults with DCD to rate themselves as being more likely to experience an accident of that type and in fact they might be more at risk potentially making it a realistic judgment. However, whether this extends to accidents without an overt motor component is unclear. Within these research questions we considered the issue of co-occurrences by comparing a group with DCD and DCD plus one (or more) neurodevelopmental disorders which have not been found to have any potential road crossing difficulties (i.e., Dyslexia, Dyscalculia, DLD, etc.) to those with DCD plus one (or more) of the neurodevelopmental disorders shown to have potential road crossing difficulties (i.e., ADHD/ADD, ASD, LD).

## Materials and Methods

### Participants

Participants were recruited via two methods, via the author’s links on social media and via the author’s personal contacts with individuals with DCD and parents of individuals with DCD. A total of 93 adults answering for themselves completed the questionnaire, however, six of these adults indicated no DCD (or Dyspraxia) related difficulties either diagnosed or undiagnosed and as such these adults were excluded, resulting in a total of 87 adult respondents with DCD. The majority of adults, 62.1%, with DCD reported crossing roads every day. Respondent demography is summarized in Table 1. A total of 75 parents completed the questionnaire, however, four of these indicated no DCD or Dyspraxia related difficulties in their child either diagnosed or undiagnosed and three participants indicated the age of their child to be over 18 years of age and so these participants were excluded leaving a final sample of 68 parent respondents. A total of 44.1% of the parents reported their child crossed the road every day, with 33.8% reporting that their child never crossed the road (either accompanied or unaccompanied). Respondent demography is summarized in Table 1.

**TABLE 1:** Participant demographics.

	Adults	Parents
*N*	87	68
Age range	17–73 years	6–18 years
Mean age	32 years	11 years
Gender ratio	58 female, 24 male, 5 neither	16 female, 51 male, 1 neither
% from United Kingdom	82	75
DCD + ADHD (N)	20	30
DCD (N)	67	38
Previous accident (%)	21.8%	0%

### Measures

The protocol for the study conducted was preregistered and is available online at the Open Science Framework (doi10.17605/OSF.IO/HWMS5), no major change were made to this initial protocol. The questionnaire used can be found in Supplementary Material.

#### Perception of Ability

All participants were asked to compare their (or their child’s) road crossing behavior and ability to their peers, the format of these questions, i.e., comparing their behavior to other children their age comes from previous measures (Purcell, 2012; Purcell and Romijn, 2017). This section included five questions, the first two asked whether they felt they paid more or less attention or exhibited more or less risk compared to their peers, these were both measured on a five point Likert scale. The third question asked the participant to rate their confidence compared to their peers on a four point Likert scale. Finally participants were asked to state whether they felt their DCD, or their child’s DCD, changed the way they crossed the road compared to their peers, based on a yes/no answer. If they answered yes they were asked to elaborate.

#### Road Crossing Behaviors

All participants were asked about the regularity with which they, or their child, exhibited certain behaviors, these questions were taken from previous measures (Chinn et al., 2004). Participants had to state regularity on a four point Likert scale (never, sometimes, often, always). The behaviors were: forgetting to look before crossing; running across without looking; seeing a small gap and going for it; crossing before the green man appears; crossing between cars; thinking there is enough time to cross but discovering there is not; looking both ways before crossing; keeping looking the whole way across; making traffic slow down so you can cross; getting half way across and having to run; crossing where there is no view and waiting a long time before crossing.

#### General Likelihood of Accidents

This section was only completed by adults with DCD (i.e., not the parents). These participants were shown a series of pictures taken from Sissons Joshi et al. (2017) and asked whether they felt the accident shown in the picture was more, the same amount or less likely to happen to them compared to their peers, if they provided an answer of more or less they were asked to provide a justification for their answer. The pictures depicted: an accident while cycling; an accident in the bath; an accident when pouring from a kettle; an accident on a trampoline; an accident while swimming; an accident in a thunderstorm; an accident with a dog and an accident when crossing the road.

#### Demography

Parents and adults were also asked a series of demographic questions regarding themselves or their child, including the regularity with which they crossed roads and whether this was accompanied or unaccompanied, whether they had been hit by a vehicle or bicycle in the past, the types of roads they crossed most often, i.e., one-way, two-way, etc., speed limit. We also asked about chronological age and gender and any confirmed diagnoses of DCD and other neurodevelopmental disorders.

### Procedure

One questionnaire was generated using the online platform Qualtrics aimed at individuals over 16 years of age and initially asked participants to indicate whether they were answering for themselves (adult version) or their child (parent version). If they were answering for themselves (adult version) all questions were then addressed in that manner, if they were answering for their child (parent version) questions were addressed in that manner.

### Statistical Analysis

Data are consistently reported from the two types of participants (parent, adult) within each section of the questionnaire. We also included co-occurrence as an additional factor, and as such split each group into two sub-groups, those with no co-occurrences or only co-occurrences for which there is no evidence of impact on road crossing (DCD) and those with co-occurrences of ADHD, ADD, ASD or LD (neurodevelopmental disorders which have previously been linked to difficulties with road crossing; DCD + ADHD), the values of *N* for these groups are provided in Table 1. The reported perception of confidence, attention and risk was analyzed with a two-way ANOVA (group × co-occurrence), *post hoc* tests with Bonferroni correction were used where appropriate. Prior to the ANOVA, assumption tests were conducted using Levene’s test used to determine whether the data violated the assumption of homogeneity of variance and Q–Q plots were used to determine the nature of the distribution of the data. ANOVA was only conducted where data were found to meet these assumptions. A power analysis was undertaken to determine sufficient power to conduct a two-way ANOVA of this type, and assuming a medium effect size of 0.25 and a power of 0.85 a total sample size of 146 participants would be needed. Given that the sample exceeds this two-way ANOVA was undertaken. An exploratory factor analysis (EFA) was used to reduce data from the scales of reported behavior with parallel analysis used to determine the number of factors to extract. Bartlett’s test of sphericity was conducted along with KMO tests for sampling adequacy and these are reported in text. Factor scores were created by taking average scores for questions within each factor and then these were subject to regression analyses. Prior to the regression analyses being conducted appropriate assumption tests were undertaken, i.e., Q–Q plots and residual plots were used to determine whether the residuals were normally distributed, Cook’s distance was used to determine the influence of observations and variance inflation factor (VIF) values were compared to the square root of VIF to determine collinearity within the data. Our participant sample provided adequate power for this regression analysis with (Wilson Van Voorhis and Morgan, 2007) stating a number of different cut off points all of which were met. Unrealistic optimism was analyzed using Chi-squared to determine the frequency of more, same and less responses for each accident type. Prior to Chi-squared, assumption checks were carried out by checking a sufficient expected frequency count. Friedman analysis was also used to determine the number of more, same and less responses with Durbin-Conover adjustments for *post hoc* tests. In all cases an alpha level for significance was set as 0.05.

### Content Analysis

Content analysis was used to code the open responses to two parts of the question: (1) when participants described how their DCD/Dyspraxia altered their crossing behavior and (2) when stating why they felt an accident was more/less likely. In each case responses were coded by both KW and CP using published seven steps (Treadwell, 2013). An initial set of categories were developed by KW with responses then re-coded by CP. For the question asking how DCD/Dyspraxia altered crossing behavior there were 62 responses from adults and 63 from parents. Agreement between the coders was high, with coders assigning responses to the same category in 80.2% of cases, disagreements were resolved through discussions between the coders. The coding framework identified five categories: heightened awareness (including more cautious and more anxious); lowered awareness (including more risky, impulsive, distracted); motor difficulties; perceptual difficulties (such as judging speed) and not knowing. For the question asking about why an accident was deemed more or less likely there was again high agreement between coders at the initial stage (95.3% agreement). A total of eight categories were identified when a “more” response was provided: coordination difficulties; understanding cause and effect; spatial awareness difficulties; not understanding risk; impulsivity/lack of attention; lack of experience; lack of confidence and has happened to me. A total of five categories were identified when a “less” response was provided: cautious; good knowledge; no exposure; good skill and like risks.

## Results

### Reported Behaviors

We asked participants to rate how often they displayed certain behaviors. Assumption checks revealed that KMO values were all above 0.63 with an overall level of 0.78. Bartlett’s test of sphericity was *p* < 0.001 and as such this assumption was valid. An orthogonal, varimax, rotation was performed as the resulting factors were not correlated. Three factors were extracted using parallel analysis. The adopted solution explained 57% of variance, with the first factor explaining 24.9%, the second 16.8%, and the third 15.3%. All component loadings were above 0.3 and so all questions were included in the resulting solution, loadings can be found in Table 2.

**TABLE 2:** EFA loadings for the three extracted factors.

	1	2	3
Looking both ways	−0.851		
Keeping looking all the way across	−0.794		
Forget to look	0.773		
Run across without looking	0.718		
Cross between cars	0.405		
Cross with no view	0.369		
Think there is enough time but there is not		0.745	
Start crossing and then have to run		0.672	
Make traffic slow so you can cross		0.346	
Cross before the green man			0.809
See a small gap and go for it			0.672
Wait a long time			−0.610

*1 Looking behavior and visibility. 2 Timing ability. 3 Impatience at the road-side.*

The first factor includes questions which focused on looking behavior and visibility, whether an individual looks before crossing and whether they continue to look. A high scoring participant on this factor would be reporting that they often run across the road without looking, do not often keep looking while crossing, cross without good visibility of oncoming traffic, etc. Factor 2 is a measure of timing ability, thinking there is enough time when there isn’t, making traffic slow down when crossing, having to run to get across in time. A high score on this factor would indicate that a participant often shows these timing misjudgments. Factor 3 describes impatience at the roadside, a high score on this factor would indicate a participant who crosses before the green man or waits at the roadside for very little time. Hence across all factors, high scores indicate dangerous behaviors.

In order to determine what variables influence the behaviors described by those factors a regression analysis was conducted on each factor score using co-occurrence group membership and chronological age as potential predictor variables. Prior to regression analysis assumption tests were conducted. For all three factors residuals were normally distributed as determined via Q–Q plots and residual plots, Cook’s distance indicated that there was no undue influence from a small sample of the data, with the maximum value always falling well below 1 (factor 1 = 0.25, factor 2 = 0.10, factor 3 = 0.50) and VIF values were very similar to the square root of VIF indicating no collinearity issues in the data (VIF value = 1).

A significant model was found for looking behavior and visibility [*F*(2,151) = 18.5, *p* < 0.001, *R*^2^ = 0.20] and impatience at the roadside [*F*(2,151) = 4.28, *p* = 0.016, *R*^2^ = 0.05], but not timing ability. Coefficients and *p* values for all predictors for each regression analysis can be found in Table 3. For looking behavior both chronological age and co-occurrence were significant predictors. Where chronological age was higher we saw a reduction in dangerous looking behaviors (crossing without looking etc.) and visibility (crossing between cars). Furthermore, individuals with DCD + ADHD had higher scores on this factor and hence demonstrate more dangerous looking behaviors compared to DCD only. For impatience at the roadside, only chronological age was significant with an increase in age being related to an increase in impatience at the roadside and so an increase in risky behaviors, such as not waiting for the green man etc.

**TABLE 3:** Beta values, standard errors, *t* values and *p* values for the two significant regression models.

		Beta	SE	*t*	*p*
**Factor 1: Looking behavior and visibility**					
Co-occurrence group	DCD + ADHD vs. DCD	0.315	0.108	2.92	0.004*
Age		−0.017	0.003	−4.92	<0.001*
**Factor 3: Impatience at the road-side**					
Co-occurrence group	DCD + ADHD vs. DCD	0.025	0.125	0.201	0.841
Age		0.012	0.004	2.92	0.004*

Significant effects are indicated by an *.

### Perceptions

**FIGURE 1:**
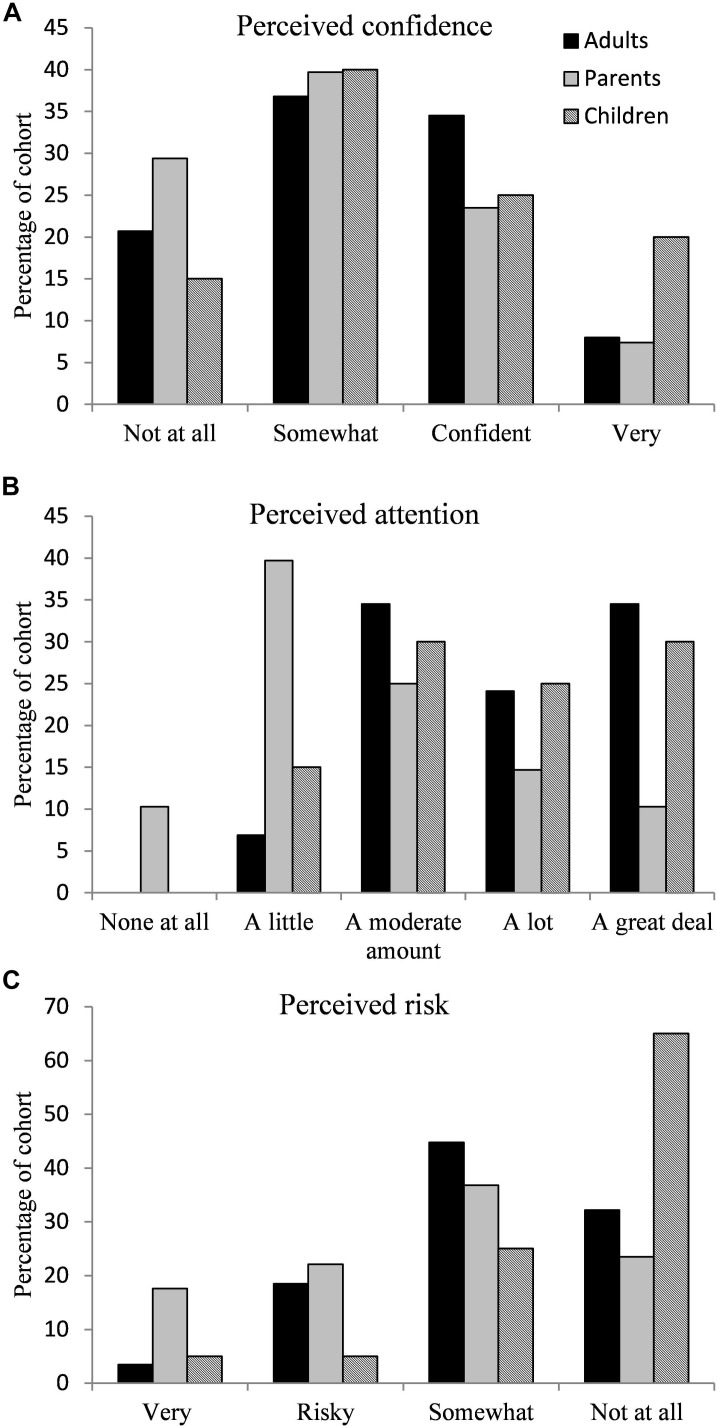
An illustration of the percentage of responses to the questions asking about **(A)** confidence in road crossing skill, **(B)** attention paid when crossing the road, and **(C)** risk taking behavior.

Three questions focused on the perception of attention, risk and confidence when crossing the road. The percentage with which confidence, attention and risk was reported can be found in Figure 1. Only 42.5% of adults with DCD and 30.9% of parents of children with DCD rated themselves or their children (parents) as confident or very confident. Furthermore, 6.9% of adults with DCD stated they paid no or little attention while crossing the road while this was much higher for the parents of children with DCD (50%). Finally, 11% of adults with DCD stated their behavior at the roadside was very risky or risky while this figure was 19.8% of parents. Two-way ANOVAs (group × presence of co-occurrence) were carried out for confidence, attention and risk separately. All of the scales met the assumption of homogeneity of variance with Levene’s test being non-significant (confidence *p* = 0.88, attention *p* = 0.50, and risky behavior *p* = 0.16). In addition, all of the scales met the assumption of normal distribution which was determined via Q–Q plots. No significant group or co-occurrence differences were found for confidence. For attention and risky behavior a significant effect of group was found [*F*(1,151) = 33.29, *p* < 0.001, η_*p*_^2^ = 0.18 and *F*(1,151) = 6.87, *p* = 0.010, η_*p*_^2^ = 0.04]. For both scales, this difference was due to parents stating less attention was paid and riskier behavior was apparent compared to the adults. In addition, a main effect of co-occurrence was found for attention [*F*(1,151) = 6.96, *p* = 0.044, η_*p*_^2^ = 0.04] and risky behavior [*F*(1,151) = 5.23, *p* = 0.024, η_*p*_^2^ = 0.03]. In both cases this was due to participants with DCD + ADHD showing significantly less attention paid and greater risky behavior compared to those with DCD. No significant interactions were observed for any of the three scales (*F* < 1). Data can be found in Table 4.

**TABLE 4:** Average responses for each group split across the co-occurrence groups.

		Confidence	Attention	Risk
		1–4 scale	1–5 scale	1–4 scale
Adult	DCD	2.34 (0.11)	3.94 (0.13)	3.07 (0.11)
	DCD + ADHD	2.15 (0.20)	3.60 (0.23)	3.00 (0.20)
Parent	DCD	2.08 (0.15)	3.03 (0.17)	2.95 (0.15)
	DCD + ADHD	2.10 (0.16)	2.40 (0.19)	2.30 (0.16)

*Standard error is given in brackets.*

When asked whether they believed their motor difficulties meant that they crossed the road differently to their peers, 79.1% of adults with DCD (80% of the DCD and 75% of the DCD + ADHD group) answered yes, while 92.6% of the parents of children with DCD (92% of the DCD and 93% of the DCD + ADHD group) indicated that their child’s motor difficulties meant they crossed the road differently to their peers. In each case the number of individuals with co-occurrences who answered yes to this question is equivalent to the proportion of the overall cohorts with co-occurrences, therefore, there is an equal representation and no further analysis of those with/without co-occurrences was made as this questions specifically asked about DCD. For those who answered *yes* to this question they were then asked how their motor difficulties changed the road crossing task for them. The categories from the content analysis and the frequency with which participants gave them as reasons can be found in Table 5. All groups reported perceptual difficulties as a key difference in their/their child’s road crossing skill compared to peers. Heightened awareness was cited by both adult groups (DCD and DCD + ADHD) and parents of children in the DCD group. In addition, lowered awareness was commonly cited in the parent groups and in the DCD + ADHD adult group. Lowered awareness was the most commonly cited reason given by parents of the DCD + ADHD group. Some indicative quotes are provided in Table 6.

**TABLE 5:** Frequency of responses to the content analysis categories regarding how motor difficulties changed the road crossing experience for adults with DCD and parents of children with DCD.

Category	Type of comment	Adults		Parents	

		DCD	DCD + ADHD	DCD	DCD + ADHD
Heightened awareness	More cautious	43.8%	33.3%	17.1%	10.7%
	More anxious				
Lowered awareness	More risky	14.6%	33.3%	28.6%	50.0%
	Not paying attention				
	Oblivious to rules				
Motor difficulties		6.3%		5.7%	10.7%
Perceptual difficulties	Judgment of speed/distance	35.4%	26.7%	45.7%	25.0%
I don’t know/response uncodable			6.7%	2.9%	3.6%

*Blank cells indicate no response fell within that category for that group.*

**TABLE 6:** Quotes regarding reasons as to why road crossing was perceived to be affected by DCD.

Group	Category	Quote
Adults	Judgment of speed/distance	My timing for crossing, and misjudgment of car distances is always way off
	Judgment of speed/distance	Can’t judge distance/speed so juts have to guess a lot of the time
	More cautious	Being far, far more cautious
	More cautious	I wait longer, and only cross if I know that I’m totally safe
Parents	Judgment of speed/distance	He has trouble judging how far away the vehicle is and how long he might have to cross the road
	Judgment of speed/distance	No road sense, unable to judge distance and speed of traffic
	Not paying attention	My son is 9 and I have no little faith in his ability to safely cross roads unassisted so I walk him to and from school every day. In places where I do allow him to cross without guidance I have seen him cross without looking, stumble into the road, be unaware he is on a road, cross between park cars and walk out into traffic.

### Unrealistic Optimism

**FIGURE 2:**
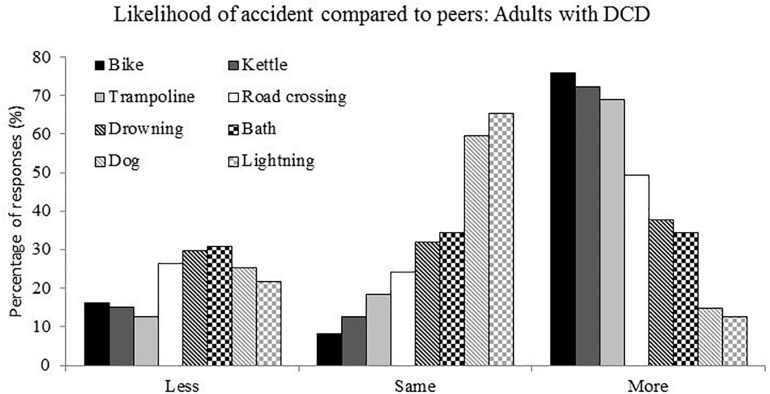
Frequency of responses for the question asking about the likelihood, compared to peers, of non-road crossing accidents.

The adults with DCD were asked to rate whether they felt an accident, depicted by an illustration was more likely, less likely or had the same likelihood to happen to them. The percentage of responses can be found in Figure 2. Chi-squared analysis revealed a difference in responses for all but accidents in the bath and drowning accidents. With accidents on the bike, on the road, with a kettle and on the trampoline being perceived as more likely (Bike χ^2^ = 71.66, *p* < 0.001, road χ^2^ = 10.21, *p* = 0.006, kettle χ^2^ = 59.86, *p* < 0.001, trampoline χ^2^ = 50.14, *p* < 0.001), accidents with a dog or with lightning (dog χ^2^ = 28.76, *p* < 0.001, lightning χ^2^ = 41.66, *p* < 0.001) being perceived as the same likelihood and bath and drowning accidents not showing a significant difference (bath χ^2^ = 0.21, *p* = 0.90, drowning χ^2^ = 0.89, *p* = 0.639). The assumptions of Chi-squared were met with expected frequencies greater than 5 in all cases and cases independent of each other.

In order to consider the frequency with which participants stated “more,” “same” or “less” the count of each of these responses was taken across the eight accident types. Giving, for each participant, a score out of 8 for each response type, as this produced ordinal data a non-parametric test was used. Friedman analysis (response type) demonstrated a significant effect across these three response types [χ^2^(2) = 29.2, *p* < 0.001] with the “more” response given significantly more often than the “same” response and the “same” response given significantly more often than the “less” response. Finally, the percentage of “more” responses across the eight accident types were calculated and compared across co-occurrence group, no significant effect was found [χ^2^(1) = 1.76, *p* = 0.19].

The reasons provided for answers of an accident being more or less likely were subject to a content analysis with reasons given collated across “more” and “less” responses with different categories for the two responses. The frequency with which individuals provided these responses for each accident type are provided in Supplementary Table 1. Just considering the accidents which were perceived to be more likely (i.e., for bike, kettle, and trampoline) in adults with DCD, in all cases coordination difficulties were given as an explanation for an accident being more likely, in fact this was the most commonly cited answer. When the adults answered “less,” the response they gave typically focused on having knowledge which would protect them from such an accident.

## Discussion

This study considered the lived experience of adults with DCD and parents of children with DCD as pedestrians at the roadside. We utilized a questionnaire which allowed us to survey exhibited behavior, perceived ability and unrealistic optimism in a large sample of these groups. We also specifically focused on the role of co-occurrences in road crossing behavior. In terms of our findings in relation to road crossing, behaviors were grouped into three factors: looking behavior and visibility, timing ability and impatience. Looking behavior and impatience varied with age, both showing a decrease in risk as age increased. Furthermore, participants in the DCD + ADHD group showed a greater level of risk in the looking behavior and visibility factor.

Previous evidence has shown us that children with DCD (Purcell et al., 2017) and children with ADHD (Clancy et al., 2006; Stavrinos et al., 2011) seemingly choose temporal crossing gaps which are too short to ensure a safe crossing or that children with DCD wait so long for a unnecessarily big crossing gap (Purcell et al., 2011). We also see children with ADHD being less concerned about risk (Farmer and Peterson, 1995) and children with LD being less able to judge the safety of crossing places (Anastasia, 2010). Finally, previous research has suggested that children with ASD may misunderstand rules of signalized crossings (Josman et al., 2008) and adults with ASD may show different eye gaze patterns when crossing (Earl et al., 2016; Cowan et al., 2018). These behaviors described in these previous findings show clear similarities to the factors from our analysis with *looking behavior and visibility* linking to eye gaze behavior (potentially atypical in ASD), identifying safe crossing places (potentially atypical in LD), and concern about risk (potentially atypical in ADHD). *Timing ability* links to choosing appropriately sized crossing gaps (potentially atypical in DCD and ADHD) and *impatience* links with waiting for a long time to cross (potentially atypical in DCD), understanding the rules of signalized crossings (potentially atypical in ASD) and concern about risk (potentially atypical in ADHD). In this way we can map previous findings in groups of children and adults with single neurodevelopmental disorders to those in the current study where we’ve considered children and adults with DCD and co-occurrences and compared these groups to each other rather than a typical group. What our findings demonstrated is that, across these factors only *looking behavior and visibility* differs between our co-occurrence groups, with those participants with DCD alongside ADHD, LD or ASD showing riskier behaviors. This supports previous research which has identified looking behavior and visibility as atypical among children and adults with ADHD, ASD and LD and from our data it would seem that these co-occurrences result in riskier behavior in individuals with DCD compared to those without those co-occurrences. In contrast, for timing ability and impatience these co-occurrences (ADHD, LD, ASD) do not impact on these areas over and above DCD. Therefore, previous laboratory based findings appear to be supported by the lived experiences, suggesting a level of awareness amongst these groups which might mitigate their risk at the roadside.

The elevated “risk” and lowered “attention” which is encapsulated within looking behavior and visibility in the DCD + ADHD group is also reflected where respondents were asked to report their perceptions of their behavior at the roadside, demonstrating that as well as reporting these behaviors these individuals (or their parents) are aware of the risky nature of some of the road crossing decisions which are made. Specifically, parents of children with DCD reported that their children paid less attention at the roadside and exhibited risky behavior. Furthermore, parents of children in the DCD + ADHD group (with co-occurrence of ADHD, ASD and/or LD) were reported to demonstrate the most risk and least attention at the roadside. It is worth noting that in Purcell and Romijn (2017) the primary school aged participants with DCD self-reported that they very much paid attention when crossing the road and didn’t often take risks. The differences between these two findings could be because participants in the Purcell and Romijn (2017) study underwent a selection process with some participants excluded due to IQ or co-occurrence, whereas the parents of those children would have remained in the current study. The differences could also be due to children with DCD not recognizing their inattention and risky behavior at the roadside, whereas their parents do. As well as asking about risk and attention, we also asked about confidence. Less than half of the adults with DCD and parents of children with DCD rated themselves or their children (parents) as confident or very confident. Again, this is in contrast to Purcell and Romijn (2017) where none of the primary school aged participants in either the DCD or TD group rated themselves as “not at all confident” and it would seem therefore, that the population in the current study are less confident than those included previously, again this could be an effect of asking parents rather than children or an effect of the more diverse nature of the sample in the current study. This type of self-reporting of perception or asking parents to report for children has not been done in populations with ADHD, ASD or LD and so comparisons with these groups cannot be made. This study grouped those without a co-occurrence and those with co-occurrences not known to cause issues at the road-side (Dyslexia etc.), however, it is not that we know that neuro-developmental disorders such as Dyslexia do not cause difficulties at the road-side, more that there is no evidence that they do. Future research is needed to pick this apart.

A key novel element of the current study was that we asked participants whether they felt their motor difficulties (adults) or their child’s motor difficulties (parents) changed the way they or their child crossed the road. Both adults with DCD (79.1%) and parents of children with DCD (92.6%) overwhelmingly reported that their motor difficulties impacted upon their road crossing, and this was regardless of co-occurrence. Although we need to be slightly cautious, due to the self-selecting nature of the current study, this does suggest that this is an area which needs careful consideration in further research and any specific remediation. When asked why, participants provided a range of reasons, only some of which were specifically focused on the motor aspect of road crossing. All groups cited perceptual difficulties as a barrier to road crossing which sits well with the literature which has highlighted this as a potential source of error when crossing the road in children with DCD (Purcell et al., 2011, 2012, 2017) and in children with ADHD (Clancy et al., 2006; Stavrinos et al., 2011). The current study extends these findings into adulthood and also highlights that parents and adults are fully aware that this is an issue. Both adult groups and the parent DCD group also cited heightened caution, which is a factor seen in some simulated road crossing studies where primary school aged children with DCD were willing to wait up to 11 s for a “safe” crossing gap when presented with a simulated single vehicle on a straight stretch of road (Purcell et al., 2011). An interesting question is *why* an adult with DCD might show caution, the current data doesn’t give us an insight into why caution is shown but it may be that they have experience of making poor road crossing decisions or struggling to safely cross the road and so have learnt to be cautious, this may explain why caution was not as commonly cited by the parents. Caution or anxiety has not previously been considered in other neurodevelopmental disorders, but the evidence from our study does not suggest that ADHD, ASD or LD consider these as a self-reported reason for difficulties with road crossing. The final factor which was commonly cited was lowered awareness (being more risky, more impulsive, more distracted), all but the adults in the DCD group cited this and it was the most common reason cited by parents in the DCD + ADHD group. It is unsurprising that where there is a majority of individuals with ADHD (regardless of other co-occurrences) impulsivity or lack of attention is cited as issues given that these characteristics are the hallmarks of ADHD. The citing of lowered awareness as a factor by parents of children without ADHD (in the DCD group) may simply be a consequence of childhood that has also been cited in studies of typically developing children at the roadside (Dunbar et al., 2001).

The final aim of this paper was to explore the issue of unrealistic optimism in adults with DCD. Anecdotal evidence would suggest that these adults are far more at risk of having very minor accidents, walking into objects, dropping things, tripping over, etc. However, how they perceive the likelihood of these accidents is unclear, i.e., do they show unrealistic optimism, i.e., the “it won’t happen to me mentality,” that we see in typically developing adults. For adults with DCD we see no evidence of unrealistic optimism, in contrast adults with DCD tended to report that they are more likely to experience an accident of any type compared to their peers, and this is regardless of their co-occurrence status. If fact adults with DCD stated they were more likely to have accidents which had a clear motor component, i.e., falling off a bike, a road traffic accident, falling from a trampoline or spilling water from a kettle and for these accident types they commonly cited “coordination difficulties” as the reason they were perceived as more likely. The literature focusing on unrealistic optimism in adults does explore mediating factors (Helweg-Larsen and Shepperd, 2001) with prior experience of an accident increasing risk estimates resulting in less optimistic bias (Helweg-Larsen and Shepperd, 2001). The findings of the current study point toward experience with accidents in the past mediating “unrealistic optimism” although it is difficult to draw firm conclusions about this as we didn’t collect data on occurrence of accidents of specific types, nor did we collect data from children with DCD who would, have less experience and so may still show unrealistic optimism. Interestingly the lack of differences across our co-occurrence groups suggests that disorders such as ADHD do not increase unrealistic optimism even though it has been linked to a lack of concern about risk (Farmer and Peterson, 1995). Adults with DCD are seemingly very aware of the likelihood of accidents and in some cases may over-estimate these and so may be mitigating this elevated risk with compensations, i.e., waiting a long time at the roadside, extra caution, etc.

As mentioned above one limitation to this study is that the population who completed this questionnaire were a self-selected population, i.e., those individuals concerned about crossing the road or concerned about their children crossing the road might have selected to complete the questionnaire while other individuals who were not concerned about this aspect choose not to. Although this might influence the data on the number of participants involved in accidents and the number of participants feeling that their road crossing was affected by their DCD it wouldn’t change the pattern of the responses in terms of regularity of behaviors at the roadside, perception of behavior or unrealistic optimism. A secondary limitation is that we collected self-reporting of behavior and so we cannot asses the accuracy of this reporting, however, this method of self-report has been used previously in adults and children and furthermore, determining true naturalistic behavior at the road-side in individuals with neuro-developmental disorders would be vastly time consuming.

This questionnaire study has demonstrated that road crossing skill is something that adults with DCD and parents of children with DCD consider to be affected by their motor difficulties. They report that decision making behaviors are more dangerous and this may be linked to perceptual and motor difficulties. Individuals with co-occurrences which have previously been linked to unsafe crossing behaviors (i.e., ADHD, ASD, and LD) also report a greater regularity of dangerous looking behavior (forgetting to look, running without looking) and visibility (crossing between cars, crossing when you can’t see), these adults and the parents of these children are seemingly aware of the risky nature of these behaviors. Unrealistic optimism was not an explanation for the risky behavior in adults with DCD and in fact, these adults demonstrated a clear understanding of the likelihood of accidents. Road crossing is clearly perceived as a different experience for adults with DCD and for parents of children with DCD and so should be recognized as an area in which remediation is needed for this population, with an understanding that those with specific co-occurrences show different behaviors.

## Data Availability Statement

Raw data supporting the conclusions of this article is available to view at https://doi.org/10.24384/nvwr-jc60.

## Ethics Statement

The studies involving human participants were reviewed and approved by the University Research Ethics Committee, Oxford Brookes University. Written informed consent to participate in this study was provided by the participants’ legal guardian/next of kin.

## Author Contributions

KW designed the study and questionnaire, collected the data, conducted the analyses, and wrote the first draft of the manuscript. CP co-designed the study and questionnaire, contributed to the content analysis coding and to the writing and revising of the manuscript. Both authors contributed to the article and approved the submitted version.

## Conflict of Interest

The authors declare that the research was conducted in the absence of any commercial or financial relationships that could be construed as a potential conflict of interest.
