# Supplementary material for: The Lived Experience of Crossing the Road When You Have Developmental Coordination Disorder (DCD): The Perspectives of Parents of Children With DCD and Adults With DCD
Source: Front Psychol. 2020 Nov 19;11:587042. doi: 10.3389/fpsyg.2020.587042 (PMC7710519; doi:10.3389/fpsyg.2020.587042)
Supplement: Supplementary file 1 [file Table_1.DOCX]

**Supplementary Table 1.** The percentage of responses regarding why an accident was deemed more/less likely, values represent the percentage of the group who answered more or less, not the entire cohort. N.B. road accidents are not provided here as this was addressed previously.

|  | | **ADULT** | | | | | | |
| --- | --- | --- | --- | --- | --- | --- | --- | --- |
|  |  | BATH | BIKE | DOG | DROWNING | KETTLE | LIGHTNING | TRAMPOLINE |
| MORE | Coordination difficulties | 44 | 47 | 50 | 61 | 43 |  | 71 |
|  | Not understanding cause and effect | 11 |  |  |  |  |  |  |
|  | Spatial awareness difficulties | 22 | 33 |  | 4 | 11 |  | 9 |
|  | Not understanding risk | 11 | 2 | 50 | 9 |  | 83 |  |
|  | Impulsive / lack of attention | 11 | 2 |  |  | 24 |  |  |
|  | No experience |  | 9 |  | 13 |  |  |  |
|  | No confidence |  |  |  | 9 | 3 |  | 3 |
|  | Has happened |  | 7 |  | 4 | 19 | 17 | 18 |
| LESS | Cautious | 36 | 15 | 67 | 21 | 36 | 7 | 67 |
|  | Knowledge | 50 | 8 | 28 | 11 | 55 | 80 | 11 |
|  | No exposure | 14 | 69 | 6 | 16 | 9 | 7 | 22 |
|  | Skill |  | 8 |  | 47 |  | 7 |  |
|  | Like risks |  |  |  | 5 |  |  |  |
