# Supplementary material for: The Lived Experience of Crossing the Road When You Have Developmental Coordination Disorder (DCD): The Perspectives of Parents of Children With DCD and Adults With DCD
Source: Front Psychol. 2020 Nov 19;11:587042. doi: 10.3389/fpsyg.2020.587042 (PMC7710519; doi:10.3389/fpsyg.2020.587042)
Supplement: Supplementary file 2 [file Table_2.DOCX]

**Questionnaire**

Are you answering this questionnaire for yourself or for your child?

For myself

For my child

**For adults only**

This set of questions asks about your behaviour when crossing the road **now.**

How often do you usually walk a route which requires you to cross roads?

Never

Less than once a week

1-2 times a week

3-4 times a week

Every day

When you walk do you usually go any part of the journey on your own?

Yes

No

Have you ever been hit by any sort of vehicle / bicycle when crossing the road?

Yes

No

Some adults pay a lot of attention to the road when crossing, others pay very little. How much attention do you pay when crossing the road?

A great deal

A lot

A moderate amount

A little

None at all

Some adults make risky crossing decisions, others make safe crossing decisions. How risky do you think your behaviour is when you are crossing the road as an adult?

Very risky

Risky

Somewhat risky

Not at all risky

|  | Never | Rarely | Sometimes | Often |
| --- | --- | --- | --- | --- |
| Forget to look |  |  |  |  |
| Run across without looking |  |  |  |  |
| See a small gap in traffic and 'go for it' |  |  |  |  |
| Cross without waiting for the green man |  |  |  |  |
| Cross between parked cars |  |  |  |  |
| Think you have enough time to cross safely, but a car is coming faster than you thought |  |  |  |  |
| Look both ways |  |  |  |  |
| Keep looking and listening until you are all the way across |  |  |  |  |
| Make traffic slow down to let you cross |  |  |  |  |
| Get half way across and have run the rest of the way, or turn back to avoid traffic |  |  |  |  |
| Cross at a place where you can't see both ways very well |  |  |  |  |
| Find yourself waiting a long time to cross |  |  |  |  |

Some adults are confident when crossing the road, others are not. Which statement best describes your confidence at crossing the road?

Very confident

Confident

Somewhat confident

Not at all confident

How often do you do the following things when crossing the road as an adult?

Look at the picture below to answer this question

<INSERT BATH PICTURE >

Do you think this type of accident is more likely, as likely or less likely to happen to you compared to other children? 
If you answer more or less can you say why you think this.

More likely ________________________________________________

The same

Less likely ________________________________________________

Look at the picture below to answer this question

<INSERT BIKE PICTURE >

|  |
| --- |

Do you think this type of accident is more likely, as likely or less likely to happen to you compared to other children? 
If you answer more or less can you say why you think this.

More likely ________________________________________________

The same

Less likely ________________________________________________

Look at the picture below to answer this question

<INSERT DOG PICTURE >

Do you think this type of accident is more likely, as likely or less likely to happen to you compared to other children? 
If you answer more or less can you say why you think this.

More likely ________________________________________________

The same

Less likely ________________________________________________

Look at the picture below to answer this question

<INSERT DROWNING PICTURE >

Do you think this type of accident is more likely, as likely or less likely to happen to you compared to other children? 
If you answer more or less can you say why you think this.

More likely ________________________________________________

The same

Less likely ________________________________________________

Look at the picture below to answer this question

<INSERT KETTLE PICTURE >

|  |
| --- |

Do you think this type of accident is more likely, as likely or less likely to happen to you compared to other children? 
If you answer more or less can you say why you think this.

More likely ________________________________________________

The same

Less likely ________________________________________________

Look at the picture below to answer this question

<INSERT LIGHTNING PICTURE >

|  |
| --- |

Do you think this type of accident is more likely, as likely or less likely to happen to you compared to other children? 
If you answer more or less can you say why you think this.

More likely ________________________________________________

The same

Less likely ________________________________________________

Look at the picture below to answer this question

<INSERT ROAD PICTURE >

Do you think this type of accident is more likely, as likely or less likely to happen to you compared to other children? 
If you answer more or less can you say why you think this.

More likely ________________________________________________

The same

Less likely ________________________________________________

Look at the picture below to answer this question

<INSERT TRAMPOLINE PICTURE >

Do you think this type of accident is more likely, as likely or less likely to happen to you compared to other children? 
If you answer more or less can you say why you think this.

More likely ________________________________________________

The same

Less likely ________________________________________________

Do you avoid crossing roads when you are on your own?

Yes

No

Do you think your Dyspraxia/DCD means you cross roads differently to other people who don't have Dyspraxia/DCD?

Yes

No

If yes, in what way?

________________________________________________________________

**Parents only**

Please answer the questions below with your child with Dyspraxia / DCD in mind. We are asking about your child as they are now, rather than when they were younger.

How often does your child walk some of the way to school?

Never

Less that once a week

1-2 times a week

3-4 times a week

Everyday

Has your child ever been hit by a vehicle / bicycle while crossing the road?

Yes

No

Some children pay a lot of attention to the road when crossing, others pay very little. How much attention do you think your child pays when crossing the road?

A great deal

A lot

A moderate amount

A little

None at all

Some children make risky crossing decisions, others make safe crossing decisions. How risky do you think your child's behaviour is when they are crossing the road?

Very risky

Risky

Somewhat risky

Not at all risky

Some children are confident when crossing the road, others are not. Which statement best describes your child's confidence at crossing the road?

Very confident

Confident

Somewhat confident

Not at all confident

Thinking about your child's road crossing behaviour how often do you think they do the following things when crossing the road?

|  | Never (1) | Rarely (2) | Sometimes (3) | Often (4) |
| --- | --- | --- | --- | --- |
| Forget to look |  |  |  |  |
| Run across without looking |  |  |  |  |
| See a small gap in traffic and 'go for it' |  |  |  |  |
| Cross without waiting for the green man |  |  |  |  |
| Cross between parked cars |  |  |  |  |
| Think they have enough time to cross safely, but a car is coming faster than you thought |  |  |  |  |
| Look both ways when crossing |  |  |  |  |
| Keep looking and listening until they are all the way across |  |  |  |  |
| Make traffic slow down to let them cross |  |  |  |  |
| Get half way across and then either run the rest of the way, or turn back to avoid traffic |  |  |  |  |
| Cross at a place where they can't see both ways very well |  |  |  |  |
| Find themselves waiting a long time to cross |  |  |  |  |

Do you feel your child's Dyspraxia / DCD affects their road crossing judgements?

Yes

No

If yes, in what way?

________________________________________________________________

At what age did your child start crossing the road unaccompanied by an adult?

In primary school

In secondary school

Unsure

My child does not cross the road unaccompanied by an adult

**For all participants**

Thank you for the time you have spent responding to our questions. In order to help us describe our participants, please answer the following demographic questions (if you are a parent please answer this for your child).

What gender are you / your child?

Female

Male

Non-binary

Different Identity

What is your / your child's current age?

________________________________________________________________

What is your / your child's primary diagnosis / difficulty (i.e. Dyspraxia / DCD, Dyslexia, ADHD etc).

________________________________________________________________

Do you / your child have any additional diagnoses / difficulties (i.e. Dyspraxia / DCD, Dyslexia, ADHD etc)?

________________________________________________________________

Is there any other information you would like to provide?

________________________________________________________________
